# Variations in clinical course and surgical outcomes of acute appendicitis during COVID-19 Pandemic: a multicenter cohort study

**DOI:** 10.1186/s12893-023-01933-8

**Published:** 2023-03-14

**Authors:** Carlos Eduardo Rey Chaves, Felipe Girón, Ricardo E. Núñez-Rocha, Elkin Benítez, Saralia Ruiz, Lina Rodríguez, Daniela Ayala, Carlos José Villamil, Valentina Galvis, Marco Vanegas, Mónica Gómez, Ricardo Nassar, Juan David Hernández, Danny Conde, María Gómez Zuleta

**Affiliations:** 1 Department of Surgery and Specialties, Pontificia Universidad Javeriana, Cra 6A #51A-48, 110100 Bogotá D.C, Colombia; 2 Department of Surgery, Fundación Santa Fé de Bogotá, Bogotá D.C, Colombia; 3 School of Medicine, Universidad de los Andes, Bogotá D.C, Colombia; 4 School of Medicine, Universidad del Rosario, Bogotá D.C, Colombia; 5 School of Medicine, Fundación Universitaria Juan N. Corpas, Bogotá D.C, Colombia; 6 Hospital Universitario Mayor, Méderi, Universidad El Rosario, Bogotá D.C, Colombia

**Keywords:** Appendicitis, COVID 19, Multicenter study, Outcomes, SARS-CoV-2, Surgery

## Abstract

**Background:**

COVID-19 pandemic has led to changes in the presentation and treatment of surgical pathologies. Therefore, we aim to describe the influence of the COVID-19 pandemic on the clinical presentation and management of acute appendicitis (AAp) and its surgical outcomes.

**Study design:**

A multicenter cohort study with prospectively collected databases. Three high-volume centers were included and all patients over 18 years of age who underwent appendectomy for AAp were included. Multiple logistic regression and multinomial logistic regression were performed, and odds ratio, relative risk, and B-coefficient were reported when appropriate, statistical significance was reached with p-values < 0.05.

**Results:**

1.468 patients were included (709 in the pre-pandemic group and 759 in the COVID-19 group). Female patients constituted 51.84%. Mean age was 38.13 ± 16.96 years. Mean Alvarado’s score was 7.01 ± 1.59 points. Open surgical approach was preferred in 90.12%. Conversion rate of 1.29%. Mortality rate was 0.75%. There was an increase of perforated and localized peritonitis (p 0.01) in the COVID-19 group. Presence of any postoperative complication (p 0.00), requirement of right colectomy and ileostomy (p 0.00), and mortality (p 0.04) were higher in the COVID-19 group. Patients in the pre-pandemic group have a lesser risk of mortality (OR 0.14, p 0.02, 95% CI 0.02–0.81) and a lesser relative risk of having complicated appendicitis (RR 0.68, p 0.00, 95% CI 0.54–0.86).

**Conclusion:**

Complicated appendicitis was an unexpected consequence of the COVID-19 pandemic, due to surgical consultation delay, increased rates of morbidity, associated procedures, and mortality, influencing the clinical course and surgical outcomes of patients with AAp.

## Background

AAp is the most common etiology of acute abdomen worldwide and has an incidence ranging from 5.7 to 57 cases per 100,000 inhabitants per year [1, 4]. The peak incidence occurs between the late teens and early twenties, in concordance with lymph development. This incidence changes according to age, ethnicity, sex and geographical factors. The lifetime risk of developing AAp is 8.6% in men and 6.7% in women [5–7].

The management of AAp has been surgical over the years with high significant efficacy and low complication rate, and is recommended for the treatment of AAp in those cases in which there is a dilatation of more than 13 mm of the appendix, mass effect or presence of appendicolith in imaging [5, 10]. However, recent evidence from different series [8, 9] has proposed medical management for this condition in specific high surgical risk populations. [5].

Despite recommendations that non-surgical management of uncomplicated AAp could postpone surgical treatment [8, 9], it is not being taken into account that the time interval to surgery is an essential factor that can lead to the progression of the pathophysiological cascade of appendicitis [11]. Previous series [12, 13] have demonstrated the direct association between the time of symptom onset and treatment, this being a modifiable factor in relation to the risk of appendiceal perforation. Thus demonstrating that the risk of rupture after a 12-h period rises to 5% after 36 h of no treatment for AAp [13]. However, others [14] show that delay in appendectomy does not increase the risk of perforation but is associated with increased surgical site infection.

During the COVID-19 pandemic, healthcare systems around the world collapsed, and the priorities around other urgent conditions changed. Moreover, concerns about increased mortality in covid patients with concomitant surgical pathologies were also present [15–17]. The Pandemic Surgery Guidance Consortium [18] recommended non-operative management of AAp and even went so far as to recommend resuming the use of gasless laparoscopy. This was based on the risk of a high case fatality rate secondary to SARS-CoV-2 pneumonia, especially after procedures involving general anesthesia, which also goes hand in hand with a decrease in the rate of hospital admissions due to the psychological effects on the population that arrive at the hospital could expose them to the pandemic pathogen [15–19].

Several studies [15, 19–23] have focused on understanding what are those consequences generated by the pandemic period that led to the delay in hospital care for the treatment of AAp. The vast majority of these studies demonstrate that this global crisis directly affected hospital admission rates for AAp in the general population, leading to delayed surgical management, with higher rates of complicated appendicitis in adults as Akbulut et al. evidenced [23], as well other surgical population as pediatric patients reveals an increased rate of complicated AAp in pediatric patients with more severe disease and suboptimal outcomes [21]. This was also evidenced in the elderly population, shown by the increased rate of conversion to laparotomy [20].

Considering that in the Latin-American population the impacts in terms of outcomes of the COVID-19 pandemic period in the management of AAp and its respective complications have not been described, this observational multicenter cohort study was developed with the aim of analyzing the clinical course and surgical outcomes of AAp and comparing these results in pre-pandemic and pandemic periods.

## Methods

### Study population

**Fig. 1:**
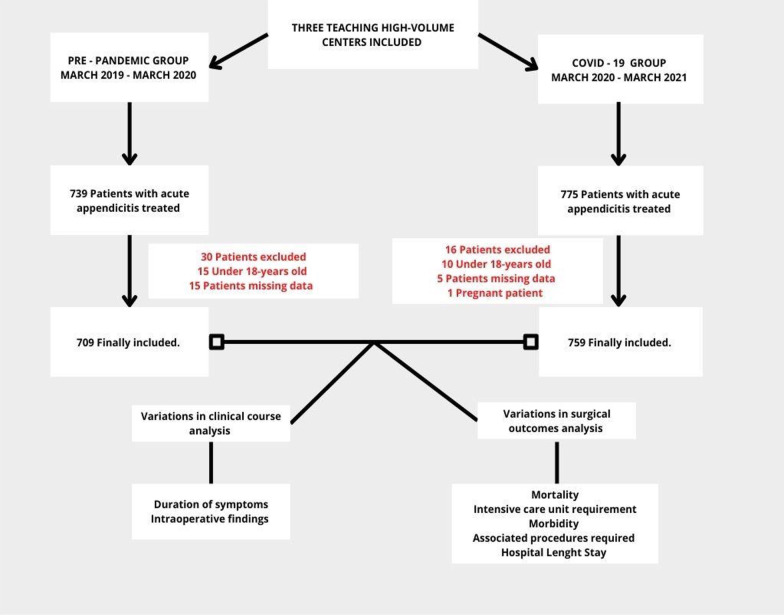
Study design

With the Institutional Review Board’s approval (DVO005 1864-CV1509) and following Health Insurance Portability and Accountability Act (HIPAA) guidelines, a multicenter cohort study with prospectively collected databases was conducted. Three high-volume centers were included and all patients over 18 years of age who underwent appendectomy for AAp were included. Cohorts were defined according to the first case of COVID-19 in Colombia as the “Pre-Pandemic group” (Patients between March 2019 and March 2020) and COVID-19 group (Patients between March 2020 and March 2021) (Fig. 1). Patients with non-operative management for AAp, missing data, no > 30 days follow-up, and pregnant patients were excluded. Ethical compliance with the Helsinki Declaration, and current local legislation on research.

### Data management

Demographic and clinical characteristics include gender, age, weight, presence of any comorbidity such as arterial hypertension, chronic obstructive pulmonary disease (COPD), chronic renal impairment, type 2 diabetes mellitus (T2DM), duration of symptoms, and Alvarado classification. As well, serum tests such as white blood cell count, neutrophil proportion, and C reactive protein were analyzed. The use of pre-operative image tests such as abdominal sonography, computed tomography, or magnetic resonance, and the type of surgical approach (open, laparoscopic, or converted) were evaluated. In terms of postoperative outcomes, surgical and medical morbidity were evaluated, as the requirement of additional intraoperative findings and requirement of additional procedures. Mortality rates, intensive care unit (ICU) requirements and hospital length of stay were analyzed.

### Statistical analysis

Descriptive statistics of all study parameters were provided according to the nature of the variable. The distribution of the variables was assessed according to the Kurtosis/Skewness test. Continuous variables were summarized by means or medians and standard deviation or interquartile ranges according to their nature and distribution. Categorical data were summarized by their frequency and proportion. Cohort analysis includes independent associations between preoperative variables and surgical outcomes with the pre-pandemic/COVID-19 Group. For categorical variables Chi-Square and Fisher exact tests were performed, in cases of continuous data two-tailed t-test, Friedman test, or Mann-Whitney Wilcoxon test were performed when appropriate. For the association between continuous data, Pearson or Spearman tests were used according to variable distribution. Confounders were controlled using multiple logistic regression or multinomial logistic regression when appropriate including covariables that could change the interaction (age, gender, weight, and comorbidities), odds ratio, relative risk, and B-coefficient were reported when appropriate, for all tests statistical significance was reached when p-values were < 0.05. Data were analyzed using STATA 17 licensed version.

## Results

### Overall population analysis

#### Demographic and clinical characteristics

A total of 1.468 patients were included and divided accordingly. 709 patients were in the pre-pandemic group and 759 were in the COVID-19 group. Female patients constituted 51.84% (n = 761) of all patients. The mean age was 38.13 ± 16.96 years old; the median weight was 69.7 kg (IQR 60; 75). The majority of the population did not have any comorbidity (81.27% n = 1.193), the most frequent associated pathology was arterial hypertension in 12.81% (n = 188) of cases, followed by type 2 diabetes mellitus in 3.00% (n = 44) (Table 1).

**Table 1:** Overall population characteristics

Variable	Value
Age mean (SD)	38.13 (16.96)
Gender % (n)	
Female	51.84 (761)
Male	48.16 (707)
Weight median (IQR), Kg	69.7 (60;75)
Comorbidities % (n)	
No comorbidities	81.27 (1.193)
Arterial hypertension	12.81 (188)
COPD	1.77 (26)
Chronic Renal Impairment	1.16 (17)
Type 2 Diabetes Mellitus	3.00 (44)
Clinical variables	
Duration of symptoms median (IQR), hours	24 (12;72)
Alvarado Score mean (SD)	7.01 (1.59)
White blood cell count median (IQR) GB/mcL	14.900 (11.870;17.900)
Neutrophil proportion median (IQR) %	82 (74.4;90)
C Reactive protein median (IQR) mg/l	290.1 (8.43;374)
Pre-operative image % (n)	
No pre-operative image	61.10 (897)
Abdominal sonography	18.39 (270)
Abdominal computed tomography	20.30 (298)
Abdominal magnetic resonance	0.20 (3)

Duration of the symptomatology was evaluated, with a median of 24 h (IQR 12; 72). Alvarado’s classification of the patients was evaluated in the cohort, data were available for 1.393 (94.89%) patients. Mean Alvarado’s score was 7.01 ± 1.59 points. White blood cell counts were evaluated with a median of 14.900 (IQR 11.870; 17.900), and neutrophil percentage had a median of 82% (IQR 74.4; 90). Serum protein C reactive value median was 290.1 (IQR 8.43; 374). In most of the cases (61.10% n = 897) were no need for pre-operative image, however, when required, computed abdominal tomography was the most frequent (20.30% n = 298), followed by abdominal sonography (18.39% n = 270), abdominal magnetic resonance was preferred in only 0.20% (n = 3) of the population.

### Surgical characteristics and postoperative outcomes

Intraoperative findings of the appendix were evaluated. Most of the population (29.70% n = 436) presented fibrino-purulent, followed by hyperemic and edematous appendix (27.52% n = 404), and gangrenous in 16.49% (n = 242). Open surgical approach was preferred in the majority of the cases (90.12% n = 1.323), laparoscopic technique was used in 8.58% (n = 126) of the patients, with a conversion rate of 1.29%.

The surgical morbidity rate was 3.62% (n = 54); and the most frequent complication was superficial surgical site infection in 59.25% (n = 32) of the cases, followed by postoperative ileus in 1.29% of the population (n = 7). The non-surgical associated morbidity rate was 0.95%.

Surgical-associated procedures were required in 7.43% of the population, the most frequent was peritoneal lavage in 22 patients, followed by peritoneal drainage in 20 patients, right colectomy in 12 patients, and cecectomy in 8 patients. Intensive care unit (ICU) was required for 1.57% (n = 23) of the patients, with a median stay of 4 days (IQR 2; 12). The total hospital length of stay was 2 days (IQR 1; 4). Mortality rate was 0.75% (n = 11) (Table 2).

**Table 2:** Surgical characteristics

Variable	Value
Intraoperative findings %(n)	
Appendix hyperemic and edema	27.52 (404)
Appendix fibrino-purulent	29.70 (436)
Gangrenous appendix	16.49 (242)
Perforated appendix	8.86 (130)
Localized peritonitis	12.40 (182)
Generalized peritonitis	4.90 (72)
Faecal peritonitis	0.14 (2)
Surgical approach % (n)	
Open	90.12 (1.323)
Laparoscopic	8.58 (126)
Converted	1.29 (19)
Surgical morbidity % (n)	
No complication	96.32 (1.414)
Superficial Surgical site infection	2.18 (32)
Deep surgical site infection	0.41 (6)
Organ-space site infection	0.34 (5)
Evisceration	0.14 (2)
Intestinal fistula	0.14 (2)
Postoperative ileus	0.48 (7)
Medical morbidity %(n)	
No complication	99.05 (1.453)
Pneumonia	0.55 (8)
Pulmonary embolism	0.20 (3)
Deep venous thrombosis	0.20 (3)
Associated surgical procedure	
No associated procedure	92.57 (1.359)
Cecectomy	0.54 (8)
Right colectomy	0.82 (12)
Ileostomy	0.07 (1)
Surgical lavage	1.50 (22)
Umbilical hernia repair	0.68 (10)
Adhesion release	0.61 (9)
Peritoneal drainage	1.36 (20)
Partial omentectomy	0.54 (8)
Retroperitoneal drainage	0.68 (10)
Phlegmon liberation	0.61 (9)
Postoperative characteristics	
Intensive care unit requirement % (n)	1.57 (23)
Intensive care unit stay median (IQR) Days	4 (2;12)
Hospital length of stay median (IQR) Days	2 (1;4)
Mortality % (n)	0.75 (11)

### Sub-groups cohort analysis

Mean age in the COVID-19 group was similar to the pre-pandemic period (38.8 years vs 37.81 years) with no statistical significance (p 0.56). In terms of gender, female patients constituted the majority of the population in the pre-pandemic group (n = 385), and in the COVID-19 groups, male patients were the most frequent (n = 383). In terms of weight, patients in the COVID-19 group have an increased rank-sum compared with the pre-pandemic period, however, with no statistical significance (p 0.07). In the pre-pandemic group, there are fewer patients with no-comorbidities compared with the COVID-19 group (n = 569 vs n = 624), with statistical significance (p 0.01).

Duration of symptoms was higher in the COVID-19 group, however, with no statistically significant value (p 0.15). White blood cell counts were slightly higher in the COVID-19 group but did not reach statistical significance (p 0.16). As well, neutrophil proportions were evaluated, with increased values in the COVID-19 group with no statistical difference (p 0.92). Pre-operative diagnostic images were less required for patients in the COVID-19 group (n = 453 vs n = 444), with an increase in the use of abdominal computed tomography in the COVID-19 group compared with the pre-pandemic group (n = 188 vs n = 110), with statistical significant value (p 0.00).

Intraoperative findings were also evaluated, in the COVID-19 group there was an increased proportion of perforated and localized peritonitis patients compared with the pre-pandemic period (n = 74 vs n = 56 and n = 109 vs n = 73) with a statistically significant value (p 0.01). Laparoscopic surgical approach was preferred in a higher proportion in the COVID-19 group (n = 95 vs n = 31), and conversion rate to open approach was higher in the COVID-19 group (n = 15 vs n = 4). Presence of any postoperative complication was higher in the COVID-19 group, with an increase in superficial surgical site infection (n = 27 vs n = 5) and postoperative ileus (n = 6 vs n = 1), with a statistically significant value (p 0.00).

Associated surgical procedures were evaluated as well. Right colectomy and ileostomy were higher in COVID-19 group (n = 10 vs n = 2, and n = 18 vs n = 2) with statistical significance (p 0.00). The requirement of ICU stay was evaluated, with a slight difference between groups, being higher in the COVID-19 group (n = 14 vs n = 9) however, it did not reach statistical significance (p 0.37). Mortality was higher in COVID-19 group (n = 9 vs n = 2) with statistical significance (p 0.04) (Table 3).

**Table 3:** Cohort analysis

Variable/Group	Pre-pandemic group (n = 709)	COVID-19 Group (n = 759)	p Value
Age mean (SD)	37.87 (0.64)	38.38 (0.61)	0.56
Gender % (n)			
Female	54.30 (385)	49.53 (376)	***0.06***
Male	45.69 (324)	50.46 (383)	
Weight rank-sum	569,847	508,399	***0.07***
Comorbidities % (n)			
No comorbidities	80.25 (569)	82.21 (624)	***0.01***
Arterial hypertension	12.97 (92)	12.64 (96)	
COPD	1.26 (9)	2.23(17)	
Chronic Renal Impairment	0.98 (7)	1.31 (10)	
Type 2 Diabetes Mellitus	4.52 (32)	4.21 (32)	
Clinical variables			
Duration of symptoms Rank-sum	509,789.5	568,456.5	0.15
Alvarado Score mean (SD)	7.08 (0.06)	6.95 (0.05)	**0.06**
White blood cell count rank-sum	531,947	546,299	0.16
Neutrophil proportion median (IQR) %	519,976	558,270	0.92
C Reactive protein median (IQR) mg/l	545,892.5	532,353.5	***0.00***
Pre-operative image % (n)			
No pre-operative image	62.62 (444)	59.68 (453)	***0.00***
Abdominal sonography	21.86 (155)	15.15 (115)	
Abdominal computed tomography	26.51 (188)	14.49 (110)	
Abdominal magnetic resonance	0 (0)	0.39 (3)	
Intraoperative findings % (n)			
Appendix hyperemic and edema	27.50 (195)	27.53 (209)	***0.01***
Appendix fibrino-purulent	30.74 (218)	28.72 (218)	
Gangrenous appendix	19.32 (137)	13.83 (105)	
Perforated appendix	7.89 (56)	9.74 (74)	
Localized peritonitis	10.29 (73)	14.36 (109)	
Generalized peritonitis	4.23 (30)	5.53 (42)	
Faecal peritonitis	0 (0)	0.26 (2)	
Surgical approach % (n)			
Open	95.06 (674)	85.50 (649)	***0.00***
Laparoscopic	4.37 (31)	12.51 (95)	
Converted	0.56 (4)	1.97 (15)	
Surgical morbidity % (n)			
No complication	98.68 (699)	94.20 (715)	***0.00***
Superficial Surgical site infection	0.70 (5)	3.55 (27)	
Deep surgical site infection	0.14 (1)	0.65 (5)	
Organ-space site infection	0.14 (1)	0.52 (4)	
Evisceration	0.28 (2)	0 (0)	
Intestinal fistula	0 (0)	0.26 (2)	
Postoperative ileus	0.14 (1)	0.79 (6)	
Medical morbidity % (n)			
No complication	99.57 (706)	98.41 (747)	0.19
Pneumonia	0.14 (1)	0.92 (7)	
Pulmonary embolism	0.14 (1)	0.26 (2)	
Deep venous thrombosis	0.14 (1)	0.26 (2)	
Associated surgical procedure			
No associated procedure	96.33 (683)	89.06 (676)	***0.00***
Cecectomy	0.56 (4)	0.52 (4)	
Right colectomy	0.28 (2)	1.31 (10)	
Ileostomy	0 (0)	0.13 (1)	
Surgical lavage	1.55 (11)	1.44 (11)	
Umbilical hernia repair	0 (0)	1.31 (10)	
Adhesion release	0.28 (2)	0.92 (7)	
Peritoneal drainage	0.28 (2)	2.37 (18)	
Partial omentectomy	0.42 (3)	0.65 (5)	
Retroperitoneal drainage	0.14 (1)	1.18 (9)	
Phlegmon liberation	0.14 (1)	1.05 (8)	
Postoperative characteristics			
Intensive care unit requirement % (n)	1.26 (9)	1.84 (14)	0.37
Intensive care unit stay median (IQR) Days	93.5	182.5	0.35
Hospital length of stay median (IQR) Days	564,100	514,146	***0.00***
Mortality % (n)	0.28 (2)	1.18 (9)	***0.04***

Bolditalic: Statistical significant value

### Statistical analysis

A logistic regression was performed including possible confounders such as gender, age, weight, and comorbidities seeking the actual relationship between outcomes and the COVID-19 pandemic. In terms of mortality, patients who present AAp in the pre-pandemic group have a lesser risk of mortality with a statistical significance value (OR 0.14 p 0.02 95% CI 0.02–0.81). Age and weight show a relationship with the outcome, however, OR shows that there is no direct relationship with the outcome (OR 0.92, 95% CI 0.85–0.99 and OR 1.1, 95% CI 1.02–1.17) (Table 4). Patients in the pre-pandemic group have a lesser relative risk of having complicated appendicitis compared with the COVID-19 group with a statistical significance value (RR 0.68 P 0.00, 95% CI 0.54–0.86). In terms of postoperative complications, patients who present AAp in the pre-pandemic group have a lesser risk of presenting superficial surgical site infection compared with the COVID-19 group, with statistical significance (Coef − 1.57, p 0.00, 95% CI − 2.54 to − 0.61). In terms of medical postoperative complications, exposure in the COVID-19 pandemic does not show a relationship with pneumonia, pulmonary embolism, or deep venous thrombosis (Table 5). In terms of associated surgical procedures, patients in pre-pandemic group have a lesser risk to require right colectomy (Coef − 1.54, p 0.04, 95% CI − 3.07 to − 0.1), peritoneal drainage (Coef − 2.19, p 0.00, 95% CI − 3.66 to − 0.72), retroperitoneal drainage (Coef − 2.21, p 0.03, 95% CI − 4.30 to − 0.13), and phlegmon liberation (Coef − 2.53, p 0.02, 95% CI − 4.69 to − 0.37) with statistical significant value (Table 6).

**Table 4:** Multiple logistic regression for mortality

Variable/Mortality	OR	p value	95% CI
Pre-pandemic group	*0.14*	*0.02*	*0.02–0.81*
Age	0.96	0.92	0.23–4.00
Weight	*0.92*	*0.03*	*0.85–0.99*
Arterial hypertension	0.46	0.42	0.07–2.99
COPD	0.60	0.67	0.05–6.20
Chronic renal impairment	1.15	0.90	0.10–12.9
Type 2 diabetes mellitus	0.81	0.87	0.06–9.81

Italic: Statistical significant value

**Table 5:** Multiple logistic regression morbidity and pre-pandemic group

Variable/Pre-pandemic group	Coefficient	p value	95% CI
Superficial Surgical site infection	*− 1.57*	*0.00*	*− 2.54 to − 0.61*
Deep surgical site infection	*− *1.67	0.12	*− *3.84 to 0.48
Organ-space site infection	*− *1.41	0.208	*− *3.61 to 0.78
Evisceration	15.76	0.99	*− *26 to 26
Intestinal fistula	*− *17.25	0.99	*− *77 to 77
Postopeartive ileus	*− *1.82	0.09	*− *3.59 to 0.30

Italic: Statistical significant value

**Table 6:** Multinomial logistic regression intraoperative findings and associated procedures and pre-pandemic groups

Variable/Pre-pandemic period	RR	p value	95% CI
Complicated appendicitis	*0.68*	*0.00*	*0.53–0.86*
Cecectomy	1.06	0.93	0.26–4.29
Right colectomy	*0.21*	*0.04*	*0.04–0.97*
Ileostomy	0.0543	0.99	0–0
Surgical lavage	1.01	0.97	0.43–2.37
Umbilical hernia repair	0.02	0.99	0–0
Adhesion release	0.29	0.12	0.06–1.42
Peritoneal drainage	*0.1*	*0.00*	*0.02–0.48*
Partial omentectomy	0.58	0.46	0.13–2.48
Retroperitoneal drainage	*0.1*	*0.04*	*0.01–0.94*
Phlegmon liberation	*0.08*	*0.02*	*0.0–0.69*

Italic: Statistical significant value

## Discussion

The World Health Organization (WHO) published [23] that by July 2022 the total number of people infected worldwide with the SARS-CoV-2 virus is 551,226,289. Of the total number of infected people, Colombia contributes 6,175,181, making it the 18th country with the highest number of infected people worldwide and occupying the number 3 position in Latin America at present [23]. Due to this increasing slope that was noticed since the arrival of the pandemic in our population, on March 18, 2020, a curfew was declared at national level secondary to the world sanitary emergency. For this reason, the associations involved in decision-making regarding health in our population, especially the local Association of Surgery, were forced to recommend the postponement of scheduled and non-urgent surgical procedures [24].

In a short period of time, the COVID-19 pandemic led to the indefinite cancellation of all elective surgeries in order to redirect resources to treat the SARS-CoV-2 pandemic [25]. The most relevant results were the severe implications for emergency surgical services and their respective patients, thus evidencing an immediate and long-term effect on these patients [26]. Related to changes in working procedures, surgical techniques, open or minimally invasive procedures, operative flow, safety measurements of work environment, and patient education [26]. For these reasons, surgical health services were forced to modify management protocols in emergency general surgery services [24]. A clear example of this are the recommendations given by surgical societies where it was recommended that in cases of uncomplicated appendicitis, non-surgical management should be provided by means of intravenous antibiotics with a subsequent change to oral antibiotics [27]. However, in cases of therapeutic failure, emergency surgery should be performed [27].

Another example of the changes regarding emergency surgery during the COVID-19 pandemic can be evidenced by the study published by Dick et al. [28] who found a 58.3% reduction in hospital admissions comparing 2019 and 2020, however, no difference was found between the demographic characteristics of the population or hospital stay. Regarding our study, Dick et al. [28] evidenced that during 2020 appendicitis cases increased from 4.3% to 18.8% (p ≤ 0.05), as did its severity. Likewise, the number of patients who underwent emergency general surgical procedures during this period escalated from 19.1% to 42.3% (p ≤ 0.05) as did the total operative time (102.4 vs 147.7 min, p ≤ 0.05) [28].

Duration of symptoms and evolution of AAp is related to the risk of perforation and increased morbidity and mortality [29]. For that reason, the timing between the onset of symptoms, medical recognition, and surgical or medical treatment is a cornerstone in the management of AAp [29–31]. According to Busch et al. [29], a delay greater than 12 h is related to perforation and increased morbidity. Regarding the influence of the COVID-19 pandemic, multiple studies evidence a longer duration of symptoms in patients treated during the pandemic period [31–33]. According to Bickel et al. [19], patients treated in the pandemic group have a mean duration of symptoms of 2.56 days compared with pre-pandemic patients with 1.71 days with a statistically significant value (p 0.001). Akin to our results, in which patients treated in the COVID-19 pandemic have a higher duration of symptoms prior to surgery. This data is related also to increased rates of complicated appendicitis during the COVID-19 pandemic evidenced by multiple studies [22, 34, 35], that show a higher proportion of patients with complicated appendicitis (38% vs 19%, p 0.00) and severe peritonitis (42% vs 15%, p 0.00) in the COVID-19 pandemic compared with the patients treated in a pre-pandemic period. Our study found statistically significant differences regarding the prevalence of complicated appendicitis between pandemic vs pre pandemic groups (29.9% vs 22.42% respectively. p 0.00). Our data also reflects that patients in the COVID-19 group have significant differences in required surgical procedures associated with appendectomy (10.94% vs 3.67 p 0.00), with specifically an increased requirement of right colectomy (1.31 vs 0.28) and peritoneal drainage (2.37 vs 0.28), thus related with the differences in complicated appendicitis rate, however, literature is scarce regarding this topic.

In terms of postoperative morbidity, Willms et al. [36] evidenced an increased risk of major postoperative complications after appendectomy in patients treated in the COVID-19 group compared with patients who underwent the same procedure in the pre-pandemic period (12.5% vs 2.7%, p 0.00), thus related with a more severe presentation of AAp during the COVID-19 pandemic. These data are not far from our results, in which we evidenced an increased rate of overall postoperative morbidity in the pandemic group (5.8% vs 1.32%, p 0.00), in our population with a specific increase in surgical site infection, also related to a higher proportion of complicated appendicitis [36].

Since the beginning of the pandemic period, there has been a growth in the literature that has been published regarding SARS-CoV-2 and its respective conditions. Among this list of publications is the work done by the COVIDSurg Collaborative [37] in which different outcomes were evaluated, the main one being 30-day postoperative mortality in patients undergoing elective or emergency surgery. The results showed that 30-day postoperative mortality in patients without SARS-CoV-2 infection was 1.5% (95% CI 1.4–1.5) and in those patients with a preoperative diagnosis of SARS-CoV-2 was 4.1%, 3.9% and 3.6% at 0–2, 3–4 and 5–6 weeks respectively. This shows that those patients who underwent surgical procedures during the concomitant infection by COVID-19 are associated with higher mortality. For this reason, it is important to investigate different pathologies such as appendicitis, where the time interval until surgery is an essential factor that can worsen the cascade of pathophysiological progression of the disease [38].

During the COVID-19 pandemic, a debate around the safety and feasibility of minimally invasive approaches such as laparoscopic procedures was proposed [39–42]. Even though some authors such as El Boghdady [42] in a systematic review show that there is no evidence of an increased risk of transmission of COVID-19 by using a laparoscopic approach [42]. Regarding AAp, there is evidence that the open approach increased their use in the pandemic period compared with pre-pandemic patients; according to the meta-analysis published by Kohler et al. [22] open procedures are higher in the COVID-19 period compared with the pre-pandemic one (8.5% vs 7.1%), controversially in our population, open approach use was lower in the pandemic group (85.50% vs 95.06% p 0.00).

Regarding mortality, in previous series from other countries where the outcomes of patients with appendicitis undergoing surgery in the pandemic period have been evaluated, no cases of mortality have been recorded, thus arguing that mortality in the COVID-19 period does not increase [22]. Similarly, in other studies, although mortality rates are recorded and although the unadjusted mortality rate in their country showed a marked increase during the COVID-19 period, no significant changes related to in-hospital mortality were found [43]. When comparing the above information with our study, we found results that differ. In our population, an overall mortality rate of 0.75% was presented for a total of 11 patients. However, when analyzing the population by subgroups, we found that mortality was higher for the COVID-19 group with a statistically significant difference (p 0.04), for a total of 2 patients in the pre-pandemic group and 9 in the COVID-19 period group. Likewise, when the respective logistic regression was performed, it showed that, in our patients, the pre-pandemic group has a lower risk of mortality with a statistically significant value (OR 0.14, p 0.02, 95% CI 0.02–0.81). This supports the hypothesis that during the pandemic period there was an increase in mortality in patients with AAp who underwent surgery in our population.

Among the limitations of our study are its retrospective nature, and the absence of a group of non-operative management during the pandemic period. However, our study includes a large sample size in a multicenter analysis of high-volume centers, with details of the influence of the COVID-19 pandemic and enhances the understanding of the pandemic impact on AAp clinical and surgical variations.

## Conclusion

Complicated appendicitis was an unexpected consequence of the COVID-19 pandemic, with a delay in surgical consultation, increased rates of overall morbidity, the requirement of associated surgical procedures, and mortality during appendectomy. COVID-19 pandemic influences the clinical course and surgical outcomes of patients with AAp. Our data could be extrapolated to future global public health emergencies in which emergency consultation will be limited.

## Data Availability

The datasets used and/or analyzed during the current study are available from the corresponding author upon reasonable request.

## Abbreviations

CT: Computed tomography
AAp: Acute appendicitis
US: Ultrasonography
MRI: Magnetic resonance imaging
HIPPA: Health Insurance Portability and Accountability Act
COPD: Chronic obstructive pulmonary disease
T2DM: Type 2 diabetes mellitus
ICU: Intensive care unit
OR: Odds ratio
IQR: Interquartile range
Coef: Coefficient
WHO: World Health Organization
